# Delta neutrophil index as an early predictor of acute appendicitis and acute complicated appendicitis in adults

**DOI:** 10.1186/s13017-017-0140-7

**Published:** 2017-07-24

**Authors:** Dong Hyuk Shin, Young Suk Cho, Gyu Chong Cho, Hee Cheol Ahn, Seung Min Park, Seung Wook Lim, Young Taeck Oh, Ji Woong Cho, Sang O. Park, Young Hwan Lee

**Affiliations:** 1 Department of Emergency Medicine, Kangbuk Samsung Hospital, Sungkyunkwan University School of Medicine, Seoul, Republic of Korea; 2 Department of Emergency Medicine, School of Medicine, Kangdong Sacred Heart Hospital, Hallym University, Seoul, Republic of Korea; 3 Department of Emergency Medicine, Sacred Heart Hospital, Hallym University School of Medicine, Anyang, Republic of Korea; 4 Department of Emergency Medicine, College of Medicine, Kangwon National University, Chuncheon, Gangwon 200-701 Republic of Korea; 5 Department of Surgery, Kangnam Sacred Heart Hospital, Hallym University Medical Center, Hallym University College of Medicine, Seoul, Republic of Korea; 6 Department of Emergency Medicine, School of Medicine, Konkuk University Konkuk University Medical Center, Seoul, Republic of Korea

**Keywords:** Appendicitis, Complicated appendicitis, Delta neutrophil index

## Abstract

**Background:**

This retrospective study aimed to evaluate the ability of the delta neutrophil index (DNI) to predict histologically normal appendicitis preoperatively and to differentiate between simple and complicated appendicitis.

**Methods:**

The data from 650 patients were divided into positive and negative appendectomy groups (histologically normal appendicitis). The patients in the acute appendicitis group were further sub-divided into simple and complicated appendicitis groups.

**Results:**

The DNI was significantly higher in the positive group than in the negative appendectomy group (0.4 vs. −0.4, *p* < 0.001) as well as in the complicated group compared with that in the simple appendicitis group (1.2 vs. 0.3, *p* < 0.001). The DNI independently predicted a positive appendectomy and an acute complicated appendicitis in multivariate logistic regression analysis [odds ratio (OR) 2.62, 95% confidence interval (CI) (1.11~6.16), *p* = 0.028 and odds ratio (OR) 4.10, 95% confidence interval (CI) (2.94~5.80), *p* < 0.001]. The optimum cut-off for a positive appendectomy and acute complicated appendicitis were 0.2 [area under curve (AUC) 0.709] and 0.6 (AUC 0.727).

**Conclusions:**

We suggest that obtaining a preoperative DNI is a useful parameter to aid in the diagnosis of histologically normal appendicitis and to differentiate between simple and complicated appendicitis.

## Background

Acute appendicitis is the most common cause of acute abdominal pain requiring urgent surgical intervention in an emergency department (ED) [1, 2]. Increasing time periods between symptom onset and surgical treatment is a risk factor for a ruptured appendix [3]. Successful treatment depends on early diagnosis and prompt intervention.

Several blood tests are used to aid diagnosis of acute appendicitis. Approximately 80% of patients are known to have leukocytosis in acute appendicitis [4]. However, an elevated white blood cell (WBC) count has a low predictive value because the WBC is also elevated in up to 70% of patients with other causes of right lower abdominal quadrant pain [5]. Elevated C-reactive protein (CRP) levels are common in acute appendicitis, but studies disagree on its sensitivity and specificity [6]. A more recently suggested laboratory evaluation is the determination of the neutrophil-to-lymphocyte and platelet-to-lymphocyte ratios. However, their sensitivity and specificity is not more sensitive or specific than that of the WBC or CRP [7, 8].

Immature granulocytes are an indicator of increased myeloid cell production and are known to increase in infectious or inflammation conditions [9–14]. The delta neutrophil index (DNI) measures the fraction of immature granulocytes in the circulation and has recently been introduced as a new inflammatory marker [15–17]. The DNI is assessed by an automated blood cell analyzer, and tests required for the DNI can be performed simultaneously with the routine complete blood count (CBC) testing [9]. Recent studies have examined its ability to predict infectious conditions [9, 10, 18, 19]. In this study, we investigated the usefulness of the DNI as an early predictor of acute appendicitis and acute complicated appendicitis in adults.

## Methods

This retrospective observational study was performed on adults (≥19 years old) who underwent surgical treatment in suspicion of acute appendicitis from January 2015 to January 2016. Patients were enrolled from two tertiary teaching hospitals. We first selected all subjects above 19 years of age who had visited an ED and had received a surgical appendectomy during our study period, and then, we excluded them using exclusion criteria. The exclusion criteria were as follows: (1) age under 19 years old, (2) pregnant, (3) patients who did not undergo their initial blood test at the ED, (4) patients with a known immunologic deficiency state or hematologic disorders, and (5) patients who were being treated with a bone marrow suppressive agent. We then divided the final subjects into positive and negative appendectomy groups. Among the positive appendectomy group, subjects were further sub-divided into non-complicated (simple acute appendicitis) group and complicated (perforation, abscess, and localized or generalized peritonitis) group based on the surgical and histological findings. The initial biochemical markers obtained at the ED were compared between the positive appendectomy vs. the negative appendectomy groups and the non-complicated appendicitis vs. complicated appendicitis groups.

All of the blood test results used in this study were the first blood tests performed at the ED. For the complete blood cell count (CBC), the Unicel DxH^TM^ 800 Cellular Analysis System (Beckman Coulter, USA) was used. Leukocytosis and leukopenia were defined as a WBC ≥9.8 (×10^9)/L and a WBC <4.3 (×10^9)/L according to our hospital’s laboratory medicine department’s reference value, respectively. The neutrophil-to-lymphocyte ratio (NLR), lymphocyte-to-monocyte ratio (LMR), and platelet-to-lymphocyte ratio (PLR) were calculated by the ratios of the neutrophil count to lymphocyte count, lymphocyte count to monocyte count, and platelet count to lymphocyte count, respectively. C-reactive protein (CRP) was measured by a TBA 120FR Chemistry Analyzer (Tokyo, Japan), and the minimum reported value was 0.05 mg/dL. The DNI was obtained automatically by the ADVIA 2120i Hematology Analyzer (Tarrytown, NY, USA).

This study was conducted after approval from the Institutional Review Board (IRB) at our hospital. Written informed consent was exempted by the IRB. We conducted the study in accordance with the Declaration of Helsinki [20, 21]. To protect personal information, patient name, hospital number, date of birth, and social security number were deleted after assigning a serial number.

### Statistical analysis

The continuous variables with a normal distribution were presented as the mean and standard deviation (SD) and for those without a normal distribution were presented as medians and interquartile ranges (IQRs). The categorical variables were described with frequency (%). We compared the continuous variables by using the Mann-Whitney test and the categorical variables by the chi-square or the Fisher’s exact test, according to the expected frequency. Parameters showing significant differences between the two groups were further analyzed by multivariate logistic regression and by the receiver operating characteristics (ROC) to verify the usefulness as an independent predictor. After finding the best cut-off value, the sensitivity and specificity of that cut-off value was calculated. We used SPSS ver. 21.0 and MedCalc version 12.4 for our statistical analysis, and the statistical significance was based on a *p* value less than 0.05.

## Results

### Characteristics of the study subjects

Six hundred fifty patients who underwent a surgical appendectomy were included during the study period. Three hundred thirty three patients were males (51.2%), and the overall mean age was 33 years old. There were 35 patients in the negative appendectomy group and 615 patients in the positive appendectomy group. In the subgroups, there were 438 patients in the non-complicated appendicitis group and 177 patients in the complicated appendicitis group. The median age of the negative appendectomy group was significantly younger than that of the positive appendectomy group (27 vs. 35, *p* = 0.046). The proportion of females was higher in the negative appendectomy group than in the positive appendectomy group (62.9 vs. 48.0%, *p* = 0.086), although this difference was not statistically significant (Table 1).

**Table 1:** Demographic characteristics and hematologic markers between the negative and positive appendectomy groups

	Negative appendectomy (35)	Positive appendectomy (615)	*p*
Age (years)	27 (19~40)	35 (23~48)	0.046
Male, *n* (%)	13 (37.1)	320 (52.0)	0.086
WBC (cells/mL)	10,000 (7110~12,750)	12,740 (9850~15,500)	<0.001
NLR	3.3 (1.8~5.6)	6.7 (4~10.8)	<0.001
LMR	4.7 (3.6~5.9)	3.1 (2.1~4.6)	<0.001
PLR	118.5 (90.9~173.7)	153.1 (112.1~215.5)	0.003
DNI (%)	−0.4 (−2.1~0.2)	0.4 (−0.6~1.4)	<0.001
CRP (mg/dL)	2.89 (1.03~32.30)	7.28 (1.88~31.00)	0.121
GPS score	1 (1~1)	1 (1~1)	0.122

*WBC* white blood cell, *NLR* neutrophil-to-lymphocyte ratio, *LMR* lymphocyte-to-monocyte ratio, *PLR* platelet-to-lymphocyte ratio, *DNI* delta neutrophil index, *CRP* C-reactive protein, *GPS score* Glasgow prognostic score

### DNI to predict acute appendicitis

The median DNI values in the negative appendectomy group and the positive appendectomy group were −0.4 (−2.1~−0.2) and 0.4 (−0.6~1.4), respectively, and there was a significant difference in the DNI values between the groups (<0.001) (Table 1). Variables with a statistically significant difference between the groups were included for multivariate logistic analysis. In the multivariate logistic regression analysis, the DNI independently predicted a positive appendectomy [odds ratio (OR) 2.62, 95% confidence interval (CI) (1.11~6.16), *p* = 0.028] (Table 2). The area under curve (AUC) for the ability of the DNI to predict the presence of acute appendicitis was 0.709. The optimum cut-off for the initial DNI was 0.2, giving a sensitivity of 59.8% and specificity of 77.1% (Table 3).

**Table 2:** Multivariate logistic regression analysis of parameters for predicting a positive appendectomy

	Odds ratio	95% CI	*p*
Age	3.11	1.06~9.14	0.039
WBC (cells/mL)	2.64	1.15~6.01	0.021
NLR	1.53	0.49~4.72	0.461
LMR	0.60	0.23~1.59	0.305
PLR	1.75	0.57~5.33	0.327
DNI (%)	2.62	1.11~6.16	0.028

*WBC* white blood cell, *NLR* neutrophil-to-lymphocyte ratio, *PLR* platelet-to-lymphocyte ratio, *DNI* delta neutrophil index

**Table 3:** Receiver operating characteristics analysis of parameters for the prediction of a positive appendectomy

	AUC (95% CI)	Criterion	Sensitivity (95% CI)	Specificity (95% CI)	*p*
Age	0.592 (0.540–0.644)	>44	31.5 (27.9–35.4)	88.6 (73.3–96.8)	<0.001
WBC (cells/mL)	0.682 (0.422–0.817)	>10,500	70.9 (67.1–74.5)	65.7 (47.8–80.9)	<0.001
NLR	0.723 (0.687–0.757)	>5.7	57.2 (53.2–61.2)	80.0 (63.1–91.6)	<0.001
LMR	0.696 (0.659–0.731)	≤3.57	57.6 (53.5–61.5)	77.1 (59.9–89.6)	<0.001
PLR	0.648 (0.610–0.685)	>178.61	38.1 (34.2–42.0)	85.7 (69.7–95.2)	0.003
DNI (%)	0.709 (0.672–0.744)	>0.2	59.8 (55.8–63.7)	77.1 (59.9–89.6)	<0.001

### DNI to predict acute complicated appendicitis

The median DNI values in the non-complicated group and the complicated group were 0.3 (−1.2~1.0) and 1.2 (0.3~2.7), respectively, and there was a significant difference in the DNI values between the groups (<0.001) (Table 4). Variables with a statistically significant difference between the groups were included in a multivariate logistic analysis. In the multivariate logistic regression analysis, the DNI independently predicted acute complicated appendicitis [odds ratio (OR) 4.10, 95% confidence interval (CI) (2.94~5.80), *p* < 0.001] (Table 5). The AUC for the ability of the DNI to predict the presence of an acute complicated appendicitis was 0.727. The optimum cut-off for the initial DNI was 0.6, giving a sensitivity of 65.0% and specificity of 71.0% (Table 6).

**Table 4:** Demographic characteristics and hematologic markers between the non-complicated and complicated appendicitis groups

	Non-complicated (438)	Complicated (177)	*p*
Age (years)	34 (23~45)	39 (26~56)	<0.001
Male, n (%)	235 (53.7)	85 (48.0)	0.210
WBC (cells/mL)	12,700 (9795~15,500)	12,910 (9965~15,520)	0.510
NLR	6.6 (3.8~10.8)	7.1 (4.5~11.1)	0.190
LMR	3.2 (2.1~4.7)	2.9 (2.1~4.3)	0.350
PLR	153.2 (111.6~212.2)	152.2 (115~221.8)	0.690
DNI (%)	0.3 (−1.2~1.0)	1.2 (0.3~2.7)	<0.001
CRP (mg/dL)	5.85 (1.81~22.14)	95.78 (33.96~154.75)	<0.001
GPS score	1 (1~1)	1 (1~1)	<0.001

*WBC* white blood cell, *NLR* neutrophil-to-lymphocyte ratio, *LMR* lymphocyte-to-monocyte ratio, *PLR* platelet-to-lymphocyte ratio, *DNI* delta neutrophil index, *CRP* C-reactive protein, *GPS score* Glasgow prognostic score

**Table 5:** Multivariate logistic regression analysis of clinical parameters for predicting complicated appendicitis

	Odds ratio	95% CI	*p*
Age	2.07	2.94~5.80	0.002
DNI (%)	4.10	2.94~5.80	<0.001
CRP (mg/dL)	23.16	0.49~4.58	<0.001
GPS score	0.50	0.23~1.55	0.420

*DNI* delta neutrophil index, *CRP* C-reactive protein, *GPS score* Glasgow prognostic score

**Table 6:** Receiver operating characteristics analysis of parameters for the prediction of complicated appendicitis

	AUC (95% CI)	Criterion	Sensitivity (95% CI)	Specificity (95% CI)	*p*
Age	0.591 (0.683–0.770)	>51	35.0 (28–42.5)	84.1 (80.3–87.4)	<0.001
DNI (%)	0.727 (0.683–0.770)	>0.6	65.0 (57.5–72)	71.0 (66.5–75.2)	<0.001
CRP (mg/dL)	0.842 (0.790–0.886)	>28.48	80.0 (59.3–93.2)	81.3 (75.4–86.3)	<0.001
GPS score	0.539 (0.490–0.588)	>0	99.0 (96–99.9)	7.3	<0.001

*DNI* delta neutrophil index, *CRP* C-reactive protein, *GPS score* Glasgow prognostic score

## Discussion

In this study, we determined that the DNI was significantly higher in the acute appendicitis group than in the negative appendectomy group, and the DNI was an independent predictor of acute appendicitis in adults. The predictive value of the DNI for acute appendicitis was fair (AUC 0.709) and similar to that of NLR (AUC 0.723). Additionally, the DNI was significantly higher in the acute complicated appendicitis group than in the acute simple appendicitis group, and the DNI was an independent predictor of acute complicated appendicitis in adults. The predictive value of DNI for acute complicated appendicitis was fair (AUC 0.727) and lower than that of CRP (AUC 0.842). Since the DNI can easily be performed in the ED along with the routine CBC, it can help clinicians determine between acute appendicitis and acute complicated appendicitis in the early phase of the diagnostic process.

Other hematological parameters such as the WBC, NLR, LMR, and PLR were also significantly different between the negative and positive appendectomy groups. Interestingly, although the CRP was higher in the positive appendectomy group, the difference was not statistically significant (*p* = 0.121). The WBC was an independent variable in the multivariate analysis, but the ability to predict acute appendicitis was not reliable (AUC 0.682). The NLR showed a slightly larger AUC than DNI (0.723 vs. 0.709) but was not an independent variable in the multivariate analysis. The DNI was the only parameter with statistical significance in the multivariate analysis and AUC > 0.70 analysis. The DNI may be a better biomarker than other previously known markers, such as WBC, NLR, LMR, and PLR.

In comparison between acute simple and complicated appendicitis, only DNI and CRP were significantly different between the groups. The WBC, NLR, LMR, and PLR were not different between the simple and complicated appendicitis groups. This finding may be in-line with the fact that, in some patients, the WBC initially decreases in the circulation after appendiceal perforation [22]. Both the DNI and CRP were an independent predictor and had an AUC >0.70. Concerning CRP, it has no value in predicting acute appendicitis, but it is the most reliable biochemical marker in predicting the complications in this study result. Our study result is similar to that of a previous study, which reported that early CRP levels were not different between a normal appendix and acute appendicitis but were significantly different between simple appendicitis and perforated appendicitis [23]. It appears that CPR takes time to rise after inflammation and, therefore, has limited value in the early phase of the diagnostic process. CRP should be a more valuable predictor for complicated appendicitis than an early simple appendicitis. On the other hand, DNI is both useful in the early prediction of acute appendicitis and prediction of complicated appendicitis.

Although direct comparison is not possible in this study, the diagnostic efficiency of DNI for acute appendicitis may not be inferior to that of more complex scoring systems such as Alvarado score, Appendicitis Inflammatory Response (AIR) score, Raja Isteri Pengiran Aank Saleha Appendicitis (RIPASA) score, or Adult Appendicitis Score (AAS). The World Society of Emergency Surgery (WSES) Jerusalem guidelines stated that the Alvarado score (with a cut-off score <5) is sensitive to exclude acute appendicitis, but not sufficiently specific in diagnosing acute appendicitis and an ideal diagnostic scoring system remains an area for future research [24]. Di Saverio et al. argued that the group of “not likely appendicitis” may be too broad and need further distinction in this subgroup [25]. Kim et al. also reported consistent risk of missing cases of true acute appendicitis (32.4%) for a cut-off of Alvarado score ≥4 [26]. Although, the DNI is not a perfect diagnostic parameter for acute appendicitis (sensitivity of 60% and specificity of 77% with the cut-off value of >0.2) like various scoring systems, the DNI is much easier to obtain along with the routine CBC, so it is likely to be more applicable in clinical practice.

Another interesting finding concerning the demographic data is that negative appendectomies occurred more commonly in young female patients. This may be attributed to gynecological diseases mimicking AA. Conversely, the median age was older in acute complicated appendicitis than in acute simple appendicitis. This finding is in-line with the fact that perforation is more common in elderly than younger patients [27, 28]. Fifty-one-year-old was the optimal cut-off value in predicting complicated appendicitis, but age was not valuable as a predictor (AUC 0.591).

Our study has several limitations. First, this retrospective study was carried out without estimating an adequate sample size for adequate power. The number of subjects within the negative appendectomy group was very small (35) compared to the positive appendectomy group (615). Second, the symptom onset to blood test time interval was not considered in this study. Lastly, we do not exactly understand or have studied the pathophysiological mechanism of an increase in the DNI. However, we think this issue is beyond the scope of this clinical study.

## Conclusion

According to the results of our study, a DNI of >0.2 seems to be a reliable parameter to obtain a more accurate diagnosis of an acute appendicitis, and a DNI of >0.6 may help differentiate complicated from non-complicated appendicitis. The ability of the DNI to predict the presence of an acute appendicitis or a complicated appendicitis is only fair, and a DNI ≤0.2 does not exclude acute appendicitis nor does a DNI ≤0.6 exclude acute complicated appendicitis. However, if the patient is clinically suspected of an acute appendicitis and the DNI is more than 0.2, a prompt confirmatory study such as CT or an MRI should be considered early in the course of the diagnostic process.

## Abbreviations

AA: Acute appendicitis
AUC: Area under the ROC curve
CBC: Complete blood cell count
CI: Confidence interval
CRP: C-reactive protein
CT: Computed tomography
DNI: Delta neutrophil index
ED: Emergency department
IQRs: Interquartile ranges
IRB: Institutional Review Board
LMR: Lymphocyte-to-monocyte ratio
MRI: Magnetic resonance imaging
NLR: Neutrophil-to-lymphocyte ratio
OR: Odds ratio
PLR: Platelet-to-lymphocyte ratio
ROC: Receiver operating characteristics
SD: Standard deviation
WBC: White blood cell
